# The hen model of human ovarian cancer develops anti-mesothelin autoantibodies in response to mesothelin expressing tumors

**DOI:** 10.1186/1757-2215-4-12

**Published:** 2011-07-29

**Authors:** Yi Yu, Seby L Edassery, Animesh Barua, Jacques S Abramowicz, Janice M Bahr, Ingegerd Hellstrom, Judith L Luborsky

**Affiliations:** 1 Department of Pharmacology, Rush University Medical Center, 1735 W Harrison Street, Chicago, IL 60612, USA; 2 Department of Obstetrics and Gynecology, Rush University Medical Center, 1725 W Harrison Street, Chicago, IL 60612, USA; 3 Department of Pathology, Rush University Medical Center, 1750 W Harrison Street, Chicago, IL 60612, USA; 4 Department of Animal Sciences, University of Illinois, Urbana-Champagne, 1207 W. Gregory Drive, Urbana, IL 61801, USA; 5 Department of Pathology, University of Washington, 300 9th Ave Haborview R&T Rm 710, Seattle, WA 98104, USA

**Keywords:** Mesothelin, Mesothelin antibodies, Ovarian Cancer, Hens, Animal Model

## Abstract

**Objective:**

Study of the hen immune system led to seminal contributions to basic immunological principles. Recent studies of spontaneous ovarian cancer in the laying hen show strikingly similar tumor types and antigen expression compared to human ovarian cancer, suggesting hens would be valuable for studies of tumor immunology and pre-clinical vaccine development. Circulating mesothelin is a relatively specific marker for human ovarian cancer and autoantibodies to mesothelin were reported. We hypothesized that hen tumors express mesothelin and that circulating anti-mesothelin antibodies occur in response to tumors.

**Methods:**

Mesothelin mRNA expression was analyzed by RT-PCR in hen ovarian tumors and normal ovaries. Mesothelin protein expression was evaluated by immunohistochemistry (IHC) and two-dimensional SDS-PAGE Western blots. Anti-mesothelin antibodies were assessed by immunoassay of sera from hens with normal ovaries and with ovarian tumors.

**Results:**

Significant mesothelin mRNA expression was observed in 57% (12/21) of hen ovarian tumors but not in normal ovaries and was found predominantly in serous tumors as in humans. Mesothelin protein was detected in tumors with mesothelin mRNA by IHC and 2D Western blots, but not in normal ovaries or tumors without mesothelin mRNA. Circulating anti-mesothelin antibodies occurred in 44% (n = 4/9) of hens with ovarian tumors which express mesothelin mRNA and were not found in hens with tumors that did not express mesothelin (n = 0/5) or normal ovaries (n = 0/5).

**Conclusion:**

The results support the utility of the hen as a novel model for preclinical studies of mesothelin as a biomarker and a target for immunotherapy.

## Introduction

Study of the hen immune system led to seminal contributions to basic immunological principles [1]. Recent studies of spontaneous ovarian cancer in the laying hen suggest it would be a valuable model for studies of ovarian tumor immunology. The laying hen spontaneously develops ovarian tumors with numerous similarities to human tumors [2-8]including similar tumor histology and tumor types [5]. The incidence of tumors increases with age as in human ovarian cancer and tumors are fully progressive and in late stages metastasize to distant sites [2,9]. Hen ovarian tumors show similar alterations in gene expression profiles compared to human tumors [4]. Moreover, multiple proteins are similarly expressed in hen and human ovarian tumors [10] and include CA125 [11], Selenium Binding Protein 1 [12], COX-1 [6,13], E-cadherin [14], VEGF [15,16] and CYP1B1 [17]. In addition, we showed that similar to human ovarian cancer [18] hens produce anti-ovarian and anti-tumor antibodies in response to ovarian tumors [19]. However, antigen specific responses are unexplored in the hen model.

Mesothelin is a well characterized biomarker for ovarian cancer in human. Mesothelin is a 40 kDa cell-surface differentiation antigen that is normally expressed at low levels and is restricted to tissues such as the mesothelial cells lining some body cavities and epithelial cells of kidney, tonsil, trachea, and fallopian tube [20,21]. However mesothelin is highly expressed in ovarian cancer, mesotheliomas and to a lesser extent in other cancers such as pancreatic, lung, and stomach [21]. Increased mesothelin protein expression was reported in 70% of ovarian epithelial tumors and up to 100% of serous papillary ovarian cancer [21-28]. Indeed, mesothelin is shed into the circulation [29] and is one of a few specific serum markers for ovarian cancer [30-32]. In addition, mesothelin autoantibodies were detected in the sera of patients whose tumors were positive for mesothelin in ovarian cancer [33]. The frequently elevated expression of mesothelin in cancer cells compared to normal cells and the immune response to mesothelin [32,34-37] have led to exploration of mesothelin as a therapeutic target for ovarian cancers [26,31,38,39].

The biological function of mesothelin remains speculative. Studies of the mouse mesothelin gene show that it is not critical for development or reproduction in normal mice [40]. In ovarian cancer it is thought to have an effect on heterotypic cell-adhesion and cell-to-cell recognition and signaling by binding to another tumor antigen, CA125 (MUC16) to facilitate the cell invasiveness and metastasis [40-43].

Ovarian cancer has the highest mortality rate of the gynecological cancers. This is primarily due to a lack of symptoms and early detection tests. Therefore, the diagnosis of ovarian cancer primarily occurs at stage III/IV [44]. When ovarian cancer is detected early survival is greater than 80% [45,46], suggesting that earlier detection could significantly increase survival. The increased tissue expression and the presence of circulating mesothelin in human ovarian cancer is relatively specific and mesothelin shows promise as a specific marker and a target of immunotherapy for ovarian cancer. Efforts to understand the trajectory of biomarker expression and to validate early markers in pre-clinical studies are facilitated by the use of animal models. There are several models of ovarian cancer in rodents that were produced by genetic manipulation [9,47-50]. The observation that specific genetic alterations lead to specific histologic sub-types of ovarian tumors [48,50] is informative and is congruent with the concept that the different sub-types rise by different mechanisms. However, few of these models develop spontaneous ovarian tumors with the hallmarks of human tumors.

In order to use the laying hen as a preclinical model to study spontaneous immunological responses to ovarian tumor antigens and to investigate the potential of mesothelin as a therapeutic target for cancer vaccine, our objective was to determine if the hen expresses mesothelin, if mesothelin expression is increased in ovarian tumors and if circulating mesothelin autoantibody is associated with ovarian tumors.

## Materials and methods

### Animals

White Leghorn laying hens (n = 31, 2.5-3 years old) were kept under a controlled light regimen (14 h light: 10 h dark) with food and water provided *ad libitum *at the Poultry Research Farm of the University of Illinois at Urbana-Champaign. Egg production and mortality records were maintained on a daily basis. Hens with normal ovarian morphology and histology had ≥ 5 eggs per clutch, while those with ovarian tumors had ≤ 2 eggs per clutch. Hens were euthanized according to an Institutional Animal Care and Use Committee (IACUC) approved protocol and the presence of tumors determined by gross morphology and histology. Sera and tissues were collected and processed for histology and mRNA and protein expression determination.

### Reverse Transcriptase-Polymerase Chain Reaction (RT-PCR) and mesothelin sequencing

RNA was extracted with Trizol reagent (Invitrogen, Carlsbad, CA) as described previously [12]. The RNA concentration and quality were measured using 260/280 absorbance. Total RNA was treated with DNAse to remove genomic contamination and 1.0 μg used for first strand synthesis using High Capacity cDNA Reverse transcription kit (Applied Biosystems Inc, Foster City, CA) following the suggested protocol. 25 ng of first strand was used for each PCR reaction as template. Oligoperfect Designer software (Invitrogen; Carlsbad, CA) was used to design mesothelin (XM_414835.2) and actin (endogenous control, NM_205518.1). *Gallus gallus *primer sequences were shown in Table 1.

**Table 1:** RT-PCR primer pairs used for mesothelin and actin transcript amplification

amplification target	primer sequence, 5' - 3'	expected product size
Amplicon1	For -GGCAAAGCTAGGGAGCTTG Rev-AGGCCCAAACACAGTGTTG	772 bp
Amplicon2	For-ACCGCAGAGGATGTTAGCAA Rev-TGTGAACAGGCTGAAGGATG	251 bp
Amplicon3	For-GATGCTTTAATGAGCCTGACG Rev-GCTGAAACTTCGGCGTGAC	202 bp
actin	For-GCCCTCTTCCAGCCATCTTT Rev-TGGAGTTGAAGGTAGTTTCATGGAT	67 bp

Amplicon 1 and 3 were used for sequencing and amplicon 2 was used to measure the mRNA expression and for PCR validation. PCR consisted of initial denaturation at 94°C for 3 minutes, followed by 35 cycles (each cycle at 94°C for 30 sec, 57°C for 30 sec and 72°C for 1 minute). PCR products were visualized by staining 3% agarose gels with ethidium bromide. The PCR product was purified (QIAquick PCR purification kit; Qiagen, Valencia, CA) and was then directly sequenced at the DNA sequencing faciilty at the University of Illinois at Chicago using the ABI BigDye Terminator in a ABI 3100 Genetic analyzer (Applied Biosystems Inc, Foster City, CA) using the same primers. Sequences from amplicon 1 and 3 and several other sequences from hen mesothelin cDNA clones available at NCBI, along with the predicted hen mRNA (XM_414835.2) were used for *in silico *analysis to assemble the mesothelin mRNA sequence using the Blast and multiple alignment programs available at NCBI. The contour quantities (density of the band multiplied by the area of the band) of amplicon 2 bands in gels were measured using differential analysis module of Quantity One (BioRad, Hercules, CA). The contour quantities of actin bands were used for normalization of contour quantities of mesothelin bands.

### Two-dimensional SDS-Polyacrylamide Gel Electrophoresis (2D-SDS-PAGE) Western Blot

Snap frozen ovarian tissues collected at euthanasia were stored at -80°C until use. Tissues were pulverized in a dry ice-acetone bath and homogenized with a Polytron (Brinkman Instruments, Westbury, NY) in ice-cold Tris-sucrose buffer, pH 7.4, (40 mM HCl, 5 mM MgSO4, 0.25 M sucrose) containing 1 μl/1 mL protease inhibitor cocktail (Sigma, St. Louis, MO). The homogenate was centrifuged (1,000 × g, 10 minutes), the supernatant collected and protein was measured (Bradford protein assay kit; BioRad, Hercules, CA) with bovine serum albumin as a standard.

Three groups of ovarian tissues were selected based on the RT-PCR result, (a) ovarian tumors (n = 4) with mesothelin mRNA expression and (b) ovarian tumors (n = 4) and (c) normal ovaries (n = 4) without mesothelin mRNA expression. Tissue groups were pooled at equivalent protein concentration for 2D PAGE Western blot. 150 μg of protein was passively rehydrated into immobilized pH gradient (IPG) strips (BioRad, 5-8 NL) and isoelectric focusing was done according to the manufacturers' protocol. The IPG strip was applied to a 10% Tris-HCL SDS-PAGE gel (Biorad, Hercules, CA) and electrophoresed. The proteins in the gel were transferred to nitrocellulose membranes using a semi-dry transfer apparatus (BioRad, Hercules, CA), and after blocking (1× blocking buffer [Sigma-Aldrich, St. Louis, MO] containing 0.05% Tween) (1 hour, 22°C), the membranes were probed with mesothelin monoclonal antibody Clone 4H3 [32](1:5000, 16 hours, 4°C). After washing (3×) with TBST (TBS containing 0.05% Tween), membranes were incubated with goat anti mouse IgG conjugated with horseradish peroxidase (Jackson ImmunoResearch Laboratories, Inc. West Grove, PA) as secondary antibody (1:10,000,1 hour, 22°C). Antibody reaction was visualized with SuperSignal West dura extended duration chemiluminescence substrate (Thermo Scientific/Pierce, Rockford, IL) and images were captured with a Chemidoc imaging system (BioRad, Hercules, CA).

### Histology and Immunohistochemistry

As described previously[5], tissue fixed in 10% buffered formalin and paraffin-embedded was sectioned (5 um), and mounted on microscope slides. Deparaffinized sections were boiled in antigen unmasking solution (11 minutes, 1:100; Vector Laboratories, Burlingame, CA) and incubated in 0.3% hydrogen peroxide-methanol (20 minutes, 22°C) to block endogenous peroxidase activity. Sections were rinsed in phosphate buffered saline (PBS), blocked (2.5% normal horse serum; 20 minutes, 22°C) and incubated (2 hours, 22°C) with a monoclonal antibody to human mesothelin (1:200; clone 4H3 [32] diluted in PBS containing 1% BSA). Sections were washed in PBS and incubated (1 hour, 22°C) with a secondary antibody that is species independent (universal biotinylated anti-immunoglobulin, Vector Laboratories, Burlingame, CA) followed by (1 hour, 22°C) Avidin-horseradish peroxidase (HRP) Complex reagent according to manufacturer's instruction (Vector Laboratories, Burlingame, CA). Control staining consisted of replacing the primary antibody with PBS containing 1% BSA. The HRP was reacted with diaminobenzidine substrate (R.T.U. Vectastain Kit, Vector Laboratories, Burlingame, CA) and counterstained with Hematoxylin. Images were acquired using an Olympus Biological Microscope BX41 (Olympus, Tokyo, Japan) with a camera adaptor U-CMAD3 and were analyzed using Soft Imaging System, MicroSuite™ Biological Suite software.

### Mesothelin Antibody Immunoassay

Three groups of hen sera were tested for antibodies to human mesothelin based on RT-PCR result using the assay described by Hellstrom et al. [35]: (group 1) hens with mesothelin positive ovarian tumors (n = 9), (group 2) hens with mesothelin negative ovarian tumors (n = 5), and (group 3) normal hens (n = 5). ELISA plates were coated (100 ul of 5 ug/mL of purified mesothelin diluted in carbonate bicarbonate buffer) and incubated overnight. The plates were blocked (3% bovine serum albumin (BSA); 2 hours) and washed with PBS containing 0.1% Tween 20. Sera were serially diluted from 1:100 to 1:200 with PBS containing 3% BSA and added to each well (1 hour, 22°C); 3% BSA was used as a negative control. Mouse anti chicken IgY conjugated with horseradish peroxidase was added as the secondary antibody (1:1000, 1 hour, 22°C). The plates were washed with PBS containing 0.1% Tween20 and sureBlue TMB Microwell Peroxidase substrate was added (15 minutes). The reaction was stopped using TMB stop solution. Results were determined as optical density (OD) at 450 nm.

### Statistical analysis

A Fishers exact test was used to determine if difference between groups were significant for mesothelin mRNA expression. The difference in mean values of the optical density for each group in the mesothelin antibody immunoassay was assessed with a t test using Welch's correction.

## Results

### In silico analysis of mesothelin transcripts

**Figure 1:**
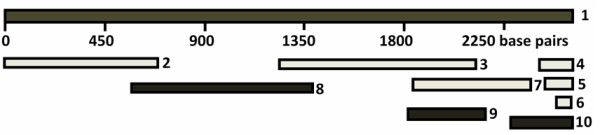
**Sequence analysis of mesothelin mRNA**. Sequence 1 is the predicted hen mesothelin mRNA (XM_414835.2). Sequences 2 to 7 are cDNA clones with the known sequences and sequences 8 to 10 are sequences from amplicon 1, 2 and 3. The sequences were aligned using BLAST (bl2seq) pair wise blast in NCBI. These results confirmed that the predicted mesothelin mRNA sequences are expressed in the hen ovary. (Sequence 1: XM_414835.2, predicted Gallus gallus similar to Mesothelin; Sequence 2: DR426891; Sequence 3: BU237188; Sequence 4: BU421473; Sequence 5: BU456162; Sequence 6: DR429030; Sequence 7: DN851245; Sequence 8: amplicon 1; Sequence 9: amplicon 2; Sequence 9: amplicon 3).

The hen genome was released in 2004. The NCBI database contains several sequence fragments for the mesothelin gene as well as a predicted mRNA sequence for mesothelin (XM_414835.2). In order to confirm that the predicted hen mesothelin mRNA sequence (sequence 1 in Figure 1) is expressed in the hen ovary, we aligned the mesothelin mRNA sequence using several sequences from cDNA clones available at NCBI Genbank: DR426891, BU237188, BU421473, BU456162, DR429030 and DN851245 (sequences 2 to 7, Figure 1) using the NCBI multiple alignment program. The resulting contig had two gaps; one gap occurred between the predicted mesothelin mRNA nucleotide positions 703 to 1233 and another short gap of 7 nucleotides occurred between positions 2357 to 2364. We designed two primer pairs to amplify PCR products to fill these two gaps and the sequences which form amplicon 1 and 3 (sequence 8 and 10 in Figure 1) were aligned perfectly with the contig we obtained previously. These results clearly showed that the predicted mesothelin mRNA sequences are expressed in chicken ovary.

### Expression of mesothelin in normal ovary and ovarian tumors

**Figure 2:**
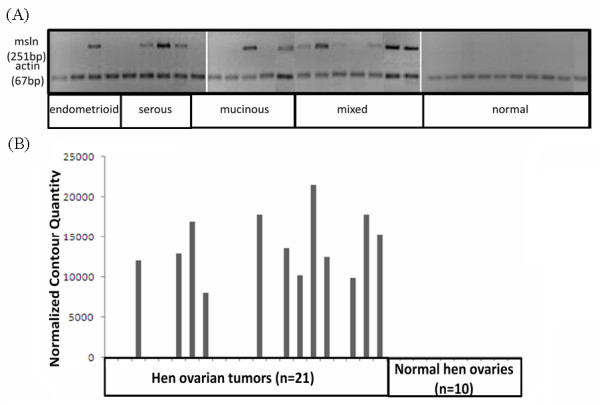
**Examples of mesothelin mRNA expression in hen ovaries containing tumors and in normal ovaries**. (A) mRNA was detected with hen specific primer based on the predicted hen mesothelin sequence. Mesothelin mRNA expression was identified in 1/4 (25%) endometrioid tumors, 3/4 (75%) serous carcinomas, 2/6 (33%) mucinous carcinomas, and 6/7 (86%) mixed histology tumors. Mesothelin was not detected in normal ovaries. (B) Measurement of mesothelin mRNA expression using differential density analysis with the loading control β-actin used as a reference. Samples are in the same order in A and B.

The mesothelin primer pair 2 was designed to produce a 251 bp product between exon 8 and exon 10. It was detected in 57% (12/21) of ovarian cancers (Figure 2A), including 75% (3/4) of serous, 33% (2/6) of mucinous, 25% (1/4) of endometrioid and 86% (6/7) of mixed histology tumors. In normal ovaries (n = 10), no mesothelin mRNA was detected in agarose gels (n = 10) (Figure 2A). The difference in mesothelin mRNA expression between normal and tumor containing ovaries estimated from normalized contour quantities of bands (Figure 2B) was significant (p < 0.0001, Fishers exact test). The 251 bp product was purified, sequenced and blasted against hen genome. The blast result confirmed that the sequence is from hen mesothelin mRNA.

**Figure 3:**
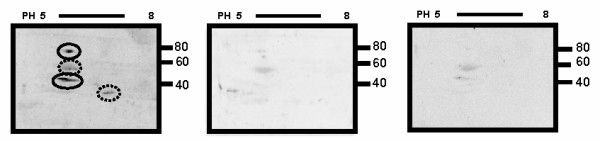
**Comparison of the expression of mesothelin protein in mRNA positive tumor ovaries (left), mRNA negative tumor ovaries (middle) and in normal ovaries (right)**. Mesothelin was detected at a pI range of 5.6 to 6.6 and molecular weights of 80 and 40 kDa (solid circles) in 2D Western blots using a monoclonal antibody to human mesothelin (clone 4H3). No mesothelin was detected in mRNA negative tumor ovaries or in normal ovaries. Some faint reactions (dotted circle) were observed in mRNA positive tumor ovaries blot and the identity of those spots remains to be determined.

Two-dimensional -SDS-PAGE Western blots of pooled mesothelin mRNA positive ovarian tumors (n = 4) showed two predominant isoelectric trails of immunoreactive proteins around 80 kDa and 40 kDa (Figure 3, left). The pI range of these trails was 5.6 to 6.6, consistent with the predicted pI of 6.6. We also observed faint reactions around 30 kD in pooled tumor ovaries. No detectable level of immunoreactive protein was found in the pooled mesothelin mRNA negative tumors (Figure 3, middle) or in the pooled normal ovarian tissues (Figure 3, right).

### Localization of mesothelin in normal ovary and ovarian tumors

**Figure 4:**
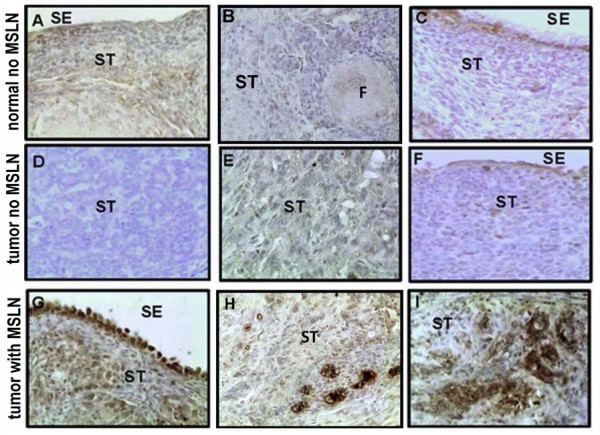
**Immunohistochemical localization of mesothelin in normal hen ovaries and hen ovaries containing tumor**. Staining was not observed in normal ovaries (A-C) in either the stromal or follicular (F) compartments or in ovarian tumors that did not express mesothelin (D-F). Mesothelin staining was observed in the ovarian surface epithelium (SE) and clusters of cells in the ovarian stroma (ST) of tumors that express mesothelin (G-I). Examples of different ovarian tumor histology are shown (D and F, advanced mucinous tumor; E, advanced endometrioid tumor; G and H, advanced serous and endometrioid mixed tumor respectively; I, advanced serous tumor). The original magnification was 40×.

Mesothelin expression was examined by immunohistochemistry in normal ovary (n = 4) and selected ovaries containing tumors (n = 6). No specific staining was seen in the normal ovary (Figure 4A and 4B) or in sections from ovarian tumors that did not show mesothelin mRNA expression (Figure 4D). However, in a very few areas, there was some weak staining in the epithelial cells and stroma of normal ovary (Figure 4C) and ovarian tumors that did not show mesothelin mRNA expression (Figure 4E and 4F). In contrast, in the tumor ovaries that exhibited mesothelin mRNA expression, intense staining was seen in surface epithelial cells, in the ovarian stroma (Figure 4G) and in aggregates of tumor cells (Figure 4I). The result showed that mesothelin expression is in small clusters of cells and appears to be at the surface of single cells (Figure 4H).

### Detection of circulating autoantibody to mesothelin

**Figure 5:**
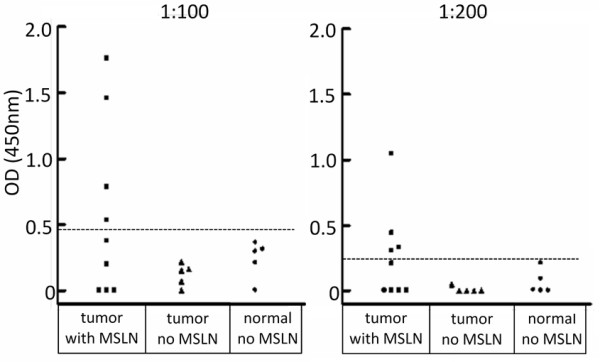
**Circulating autoantibodies to mesothelin were detected in sera of hens with mesothelin mRNA expressing ovarian tumors by immunoassay**. Sera were tested at dilutions of 1:100 and 1:200. Four of the nine hen sera with mesothelin PCR positive ovarian tumor are positive at both dilutions. None of the sera from hens without mesothelin mRNA expression had an optical density (OD) above the cutoff value for mesothelin antibody. The cutoff was determined as the mean value of normal controls plus two standard deviations above the mean. The cutoff value for positive reactions was 0.49 at 1:100 and 0.23 at 1:200.

At serum dilutions of 1:100 and 1:200, 44% (4/9) of hens with mesothelin mRNA positive tumors, but none that did not express mesothelin (tumors or normal ovaries), had OD values greater than the cutoff value for that dilution (mean of normal serum plus two standard deviation). If the tumor type is considered in the hens with circulating mesothelin antibody, three out of the four (75%) were serous carcinomas (Figure 5). The mesothelin antibody levels in hens with mesothelin mRNA positive tumors is significantly higher than in hens with negative tumors at a serum dilution of 1:100 (p = 0.039, one-tailed t test with Welch's correction) and 1:200 (p = 0.030, one-tailed t test with Welch's correction).

## Discussion

The results demonstrate for the first time that, similar to human ovarian tumors, mesothelin mRNA and protein are expressed in hen ovarian tumors and not in normal ovaries. This is also the first report of an antigen specific immune response in hens with tumors, and similar to humans serum mesothelin autoantibodies only occur in response to ovarian tumors that express mesothelin.

Mesothelin expression in human cancers has been studied extensively [21-28]. Previous studies showed that mesothelin protein frequently is expressed in human ovarian carcinomas and although there are some differences among studies, mesothelin is predominantly expressed in serous tumors [21,22], similar to the findings of this study. In our study using a chicken specific primer for RT-PCR analysis showed that mesothelin mRNA was increased significantly in hen ovarian tumors compared to normal ovaries. Similar to the human expression pattern, 75% of the hen serous ovarian tumors had mesothelin gene expression, other subtypes had varying degrees of expression but to a lesser extent than serous carcinoma and none of the normal ovaries had detectable levels of mesothelin mRNA.

Protein expression determined by Western blot analysis and immunohistochemistry was consistent with the mRNA results; mesothelin protein was expressed in tumors with mesothelin mRNA and was absent in normal ovaries. The predicted hen mesothelin sequence has 797 amino acids, a pI of 6.6 and a molecular weight of 88 kDa. Consistent with the estimated molecular size of hen mesothelin we observed two predominant trails of protein 2D Western blots; the one slightly above 80 kDa could be the full length predicted hen mesothelin, while the one around 40 kDa could be due to alternate splice variants [28,51]. By immunohistochemistry using the same anti-human mesothelin antibody as for Western blot analysis, we observed patterns of mesothelin expression that were consistent with the mRNA and protein expression. Similar to observations in human ovarian tumors [22,26,52,53], there was limited background stain in normal hen ovary and tumors that did not have mesothelin mRNA expression while there was intense staining of surface epithelial cells, tumor cells and clusters of cells in the ovarian stroma of mesothelin mRNA expressing tumors; cell surface staining was more intense than in the cytoplasm.

In addition to elevated expression of mesothelin in ovarian tumors, there was evidence of circulating mesothelin antibodies in hens with ovarian tumors. This corresponds to the reports of Hellstrom et al. [35] and Ho et al. [33] of mesothelin antibodies in humans and is also consistent with our previous report of anti-ovarian antibodies in the hen and humans with ovarian tumors [19]. It should be noted that we used human mesothelin as an immunoassay antigen since there currently are no reagents for a chicken specific anti-mesothelin immunoassay. The presence of a humoral immune reaction to ovarian tumors in the hen suggests it will be possible to use the hen for pre-clinical studies of anti-mesothelin vaccines.

In summary, the laying hen is a novel animal model of ovarian cancer because it spontaneous develops ovarian tumors with a striking histological resemblance to human ovarian tumors. The results of this study add to the growing list of ovarian cancer biomarkers that have been shown to be expressed both in hen and human. Our findings show that mesothelin gene and protein expression are elevated in chicken ovarian tumors and the results further validate the laying hen as an animal model for human ovarian cancer.

## Authors' contributions

YY performed a majority of the experiments and wrote the manuscript.

SE supervised experiments as needed, designed the PCR primer and assisted with hen mesothelin sequencing.

AB harvested the hen tissues and assisted with the immunohistochemistry.

JA supervised the ultrasound and assisted with hen selection and tissue harvesting.

JB assisted with hen selection and contributed expertise in hen physiology; hens are maintained under her supervision.

IH performed the anti-mesothelin antibody tests, provided human purified and recombinant mesothelin and contributed to the manuscript.

JL developed the concept for the study with YY, assisted with the experimental design, data interpretation and manuscript preparation and revisions.

All authors read and approved the final manuscript.

## Conflict of Interest statement

The authors declare that they have no competing interests.
